# High Safety Risk Assessment in the Time of Uncertainties (COVID-19): An Industrial Context

**DOI:** 10.3389/fpsyg.2022.834361

**Published:** 2022-04-22

**Authors:** Yuantian Zhang, M. Ridhuan Tony Lim Abdullah, Nor Hafizah bt Abd Latiff Khan, Muhammad Umair Javaid, Mohammad Nazri, Muhammad Umair Shah

**Affiliations:** ^1^Hoarded Fortune Group, Singapore, Singapore; ^2^Department of Management & Humanities, Universiti Teknologi PETRONAS, Seri Iskandar, Malaysia; ^3^Faculty of Management and Information Technology, Universiti Sultan Azlan Shah, Kuala Kangsar, Malaysia; ^4^Department of Management Sciences, Lahore Garrison University, Lahore, Pakistan; ^5^Department of Business and Accountancy, University of Malaya, Kuala Lumpur, Malaysia; ^6^Department of Management Sciences, Faculty of Engineering, University of Waterloo, Waterloo, ON, Canada

**Keywords:** downstream, COVID-19, safety behavior management, interpretive structural modeling, oil and gas industry, Malaysia

## Abstract

**Background:**

The complexities of the workplace environment in the downstream oil and gas industry contain several safety-risk factors. In particular, instituting stringent safety standards and management procedures are considered insufficient to address workplace safety risks. Most accident cases attribute to unsafe actions and human behaviors on the job, which raises serious concerns for safety professionals from physical to psychological particularly when the world is facing a life-threatening Pandemic situation, i.e., COVID-19. It is imperative to re-examine the safety management of facilities and employees’ well-being in the downstream oil and gas production sector to establish a sustainable governance system. Understanding the inherent factors better that contribute to safety behavior management could significantly improve workplace safety features.

**Objective:**

This study investigates employees’ safety behavior management model for the downstream oil and gas industry to consolidate the safety, health and wellbeing of employees in times of COVID-19.

**Methods:**

Nominal Group Technique (NGT) was first employed to screen primary behavioral factors from 10 workplace health and safety experts from Malaysia’s downstream oil and gas industry. Consequently, 18 significant factors were identified for further inquiry. Next, the interpretive structural modeling technique was used to ascertain the complex interrelationships between these factors and proposed a Safety Behavioral Management Model for cleaner production.

**Results:**

This model shows that management commitment, employee knowledge and training, leadership, and regulations contribute significantly to several latent factors. Our findings support the Social Cognitive Theory, where employees, their environment, and their behaviors are related reciprocally.

**Conclusion:**

It is postulated that identifying safety factors and utilizing the proposed model guides various stakeholder groups in this industry, including practitioners and policymakers, for achieving long-term sustainability.

## Introduction

Malaysia has the world’s 16th largest natural gas and 28th largest crude oil reserves (World-meter, 2019). That makes the downstream oil and gas industry a significant contributor to the country’s economy. The leading investor in the downstream oil and gas industry is Petroleum Nasional Berhad (PETRONAS), which is solely owned by the Malaysian government. High risks to health, safety, and the environment are the inherent characteristics of this industry. Therefore, various stakeholders must address related health, safety, and environmental concerns and take preventive actions to foster a longer-term sustainable posture. Integrating safety-related initiatives in daily operations is central to corporate sustainability practice (Nawaz et al., 2019).

According to the reports of the International Labor Organization, industrial workers around the globe may get exposed to different occupational hazards from psychological distress to chronic fatigue that put them at risk of injuries, diseases, and even deaths particularly in the context of the COVID-19 response. Safety is a major concern for the downstream oil and gas industry, as it is considered the primary source of substantial direct and indirect costs (Barling et al., 2002; Silvestre and Gimenes, 2017; Nawaz et al., 2019). Safety management issues have garnered many academics and practitioners’ interest for the longest time, over the past 65 years (Akbar and Ahsan, 2019). Typically, it is believed that organizations predominately care for economic returns, and therefore, leadership deems it essential to ensure safe and sustainable business operations (Akbar and Ahsan, 2019). However, a more holistic stakeholder engagement model has gained popularity among practitioners and academics in recent years. According to this concept, an organization has a fiduciary duty to create value for all stakeholders, including employees, suppliers, financiers, communities, and customers, with no trade-offs (Freeman, 1984). The current research focused on using the stakeholder theoretic lens in this article to address organizational sustainability. This research examines the relationship between employees in the context of safety behavior.

In the broader sustainability realm, one of the topics that have caught our attention is employees’ safety behavior at the workplace. It is opinioned that workplace-related injuries and incidents cause significant economic and emotional impacts on individuals, families, organizations, and industries in general. It harbors adverse financial consequences for the high cost of medical treatment, rehabilitation, loss of productivity, and employee morale. For that reason, management commitment is crucial in ensuring the highest standards of safety practices at the workplace (Hong et al., 2018). Prior studies highlighted a plethora of influencing factors for safe working environments. For instance, workplace environment and employees’ safety behavior are directly linked to workplace safety. Safety behavior describes how workers obey the safety rules and procedures (Luo and Wu, 2019) and how do workers consciously or unintentionally act safely or unsafely as they perform their work. Most workplace accidents and health issues are typically caused by the workers’ unsafe behavior (Cooper and Phillips, 2004; Meng et al., 2019) and mismanagement of the psychosocial work environment (Javaid et al., 2018, 2019).

Employees’ safety behavior is acclimatized through organizational leadership, safety training procedures, awareness, knowledge, and organizational commitment (Clarke, 2013; Akbar and Ahsan, 2019). Companies in the downstream oil and gas industry are known for the highest safety standards and procedures. Nevertheless, accidents occur, and remarkably, 80% of these cases are attributed to the employees’ unsafe acts and behaviors. Therefore, having the highest safety standards and procedures alone is not enough to achieve a lower Loss Time Injury Frequency Rate (LTIFR). It is a statistic that is used to benchmark the occupational health and safety performance of the company. Inevitably, safety standards and procedures critically require a model for managing safety behavior as an enabler. Thus, embracing the concept that managing desirable employees’ safety behavior at the workplace is paramount to reducing workplace accidents (Hong et al., 2018).

This study aims to develop a safety behavior management model. The safety behavior management model is a network of several behavior factors that are significant to influence safety behavior. A panel of safety experts determines the pertinent safety behavior factors in the workplace. Distinguishing the network of relationships between these factors in guiding managers, professionals, policymakers, and employees is necessary to shape and manage adequate safety behavior through collaborative interactions. Due to the complexity and dynamics of the relationships between these different factors, Nominal Group Technique (NGT) and Interpretive Structural Modeling (ISM) methods are employed (Tan et al., 2019; Yahaya et al., 2020). NGT is used to select and rank the factors. Following that, ISM is employed to analyze the network relationships among the factors that formed the model’s foundation.

## Literature Review

### Safety Behavior

Human behavior is a significant contributor to workplace accidents, as revealed in a relatively vast literature on occupational safety. Prior efforts to shape human actions to reduce workplace accidents are focused on technical aspects and engineering controls (Tomás et al., 1999). The approach did not provide satisfactory results as workplace accidents still abound. In the continuous effort to search for solutions, researchers focused on factors related to humans’ vis-a-vis behavior with the notion that unsafe human behavior caused workplace accidents (Akbar and Ahsan, 2019). Subsequent studies empirically confirmed that employees’ unsafe behavior significantly contributed to workplace accidents (Zakaria et al., 2012). Safety behavior relates to the manner the employees comply with the companies’ safety rules and procedures. This could be a conscious or an unconscious effort by the employees to either act safely or unsafely while performing their jobs (Xu et al., 2014; Arnold, 2018).

The unconscious part could be repressed feelings, memories, habits, thoughts, desires, and reactions. Ultimately, the behavior of the employees at work is crucial for their safety. Employees who comply with these safety rules and procedures tend to significantly reduce the possibility of incurring accidents (Martínez-Córcoles et al., 2011). According to the findings of a study in the petroleum refinery industry (Malott, 2016). Their research confirmed that safe behaviors in the workplace reduce the possibilities of workplace accidents. Safety behavior is generally categorized into two dimensions, namely safety compliance and safety participation. Employees’ behavior related to safety compliance at the workplace includes adherence to safety rules, standards, and regulations (Hong et al., 2018). Employees’ compliance with safety rules, regulations, and safety procedures indicates individual awareness of safety.

Occupational Safety and Health Administration (OSHA) defines safety participation as the employees’ involvement in establishing, operating, evaluating, and improving the health and safety program. Safety participation is a work culture demonstrated by the employees’ actions or activities, such as helping colleagues, attending safety meetings/talks, and volunteering to join safety programs. These initiatives promote a safe workplace environment and motivate safe behavior (Akbar and Ahsan, 2019). A safety participation culture will develop responsibility among the employees to remind each other about safe behavior and work safely (Neal and Griffin, 2006). The finding supports that employees’ participation in various safety-related programs promotes continual safety awareness (Nowicki, 2009).

### Safety Behavior Factors

The Social Cognitive Theory is used as guidance on the selection of safety behavior factors. The theory states that personal behavior and environment are reciprocally related. The theory explains that humans are motivated by themselves and are shaped by their surroundings (You, 2010). Social Cognitive Theory demonstrates that humans are not driven by internal forces and are not affected by external environments/factors. Human functionality contributes to intrinsic motivation, behaviors, and the environment while staying within a network of influences that interact reciprocally (Cui et al., 2013). Besides that, Cui et al. (2013) stated that individual employees’ safety behaviors are influenced by the organizational factors that shape their knowledge.

The study by Zin and Ismail (2012) proposed these factors that influence safety behavior: management commitment, safety leadership, training, guidelines, regulations, motivation, organizational commitment, communication, safety and health officers, and personal protective equipment. Other studies have focused on safety leadership (Cooper and Phillips, 2004), safety facilities (Martínez-Córcoles et al., 2012), safety communication (Hofmann and Morgeson, 1999), safety training (Cooper and Phillips, 2004), and safety motivation (Neal and Griffin, 2006) as organizational factors. In particular, from the standpoint of the individual employee, factors, such as safety awareness (der Wilt et al., 2008), safety commitment (Mohammadfam et al., 2016), and safety knowledge (Neal et al., 2000) are recommended. It is concluded that cumulatively the organizational, environmental, and individual-related factors at varying degrees influence the employees’ safety behavior at their workplace.

*Organizational-related factors* such as management commitment to maintaining safety, Leadership influence toward safety at the workplace, safety training provided for the employees, rules and regulation for safety at the workplace, safety and health officer, safety promotion policies, safety facilities that include personal protective equipment, and safety communication. Similarly, *the Environment related factors* are working facilities at the workplace, workplace pressure to complete the task, teamwork toward safety at the workplace. Moreover, *Individual Related Factors* are employees’ knowledge about safety at the workplace, safety communication, safety commitment, safety attitude, safety awareness, and safety motivation. Hence, from the lens of Social Cognitive Theory, these factors are considered in this study to develop a safety behavior management model.

## Methodology

This study’s main objective is to develop an Interpretive Structural Safety Behavior Management Model for employees in downstream oil and gas companies in Malaysia. This study employed the ISM technique to develop the model with expert opinions and their views via 10 experts having at least 10 years of experience from Malaysia’s downstream oil and gas industry. ISM is a well-established methodology developed by Warfield (1973) to analyze complex socio-economic systems.

The technique identifies contextual relationships among specific items, which define a problem or an issue. ISM is a computer-aided learning technique that allows groups of people or individuals to develop a structure or mapping showing interrelationships among many specific factors/elements that define an issue or problem according to a particular contextual relationship. In this study’s context, based on the decisions of a group of experts in health, safety, and environment fields, the idea about the relationships between these factors and how they are connected generates an overall structure and is demonstrated in a graphical model. The direct and indirect relationships between these factors describe the management of the safety behaviors far more precisely than the individual factor taken in isolation.

There were five (5) steps involved in the ISM technique:

### Identification and Ranking of Factors

Interpretive structural modeling starts with the identification of safety behavior factors through a literature review. A modified NGT was employed to determine the pertinent safety behavior factors (elements for the model) based on panel experts’ integrated views. NGT is a structured process used to rank major problems or issues that need to be addressed through group discussion (Potter et al., 2004). The NGT process begins with a short survey of pre-listed safety behavior factors. The pre-listed safety behavior factors define each element specific to the scope of study and guide the experts with a starting point of ideas, to begin with, thus shortening the NGT process significantly from 4 h to 90 min.

Since the proposed model was on employee safety behavior management in downstream oil and gas companies of Malaysia, the experts for the modified NGT were safety managers, safety, and health officers from the downstream oil and gas industry. These government agencies are related to safety at the workplace, namely the National Institute for Occupational Safety and Health (NIOSH), Department of Occupational Safety and Health (DOSH), Social Security Organization (SOCSO), consultants, and researchers in the area of safety behaviors. The experts have at least 10 years experience in health, safety, and environment (HSE) in the oil and gas industry.

Regarding the survey for the pre-listed safety behavior factors, the experts would vote on the importance of safety behavior elements. From the survey, factors that achieve positive consensus were considered for the next step. The experts were allowed to add additional factors if they were deemed fit for the study’s model and scope (Gorvett and Liu, 2007). Each safety behavior factor was presented, clarified, and familiarized to ensure the experts have a typical comprehension of the factors to allow appropriate judgment to the study’s context. Therefore, the experts could arrive at a consensus decision on the factors, either to be included in the model development or not. Finally, the experts would prioritize the factors assigning a preferred ranking number for each factor.

### Determine the Structural Self Interaction Matric

This step demonstrated the relationships among the elements based on the pair-wise procedure. The pair-wise relationships were based on two elements, i and j. Four symbols were used to indicate the relationship between two elements (i and j) (Mandal and Deshmukh, 1994). The description for each symbol is as follows:

• V for the relationship where element i will affect element j (when i is paired to j),
• A for the relationship where element j affects element i (when i is paired to j),
• X for mutual relations (i.e., element i and element j will affect each other), and
• O for no relationship between the elements (i.e., both elements are unrelated).

Based on contextual relationships, the Structural Self Interaction Matric (SSIM) was developed. To obtain a consensus among the respondents, SSIM needs to be discussed further by an expert panel. Based on their feedback, SSIM is finalized.

### Determine the Final Reachability Matrix

The construction of the reachability matrix was to classify the elements to different levels. This is important to develop the model structure and be interpreted at the end of the study (Lewis, 1985). This was achieved based on SSIM (Step 2) by replacing V, A, X, and O as 1 and 0, as given below. The replacement of 1s and 0s are as follows:

• “If the entry (i, j) in SSIM is V, the entry (i, j) in the target matrix becomes 1, and the entry (i, j) becomes 0,
• If the entry (i, j) in SSIM is A, the entry (i, j) in the target matrix becomes 0, and entry (i, j) becomes 1,
• If the entry (i, j) in SSIM is X, the entry (i, j) in the target matrix becomes 1 and the entry (i, j) also becomes 1, and

If the entry (i, j) in SSIM is O, the entry (i, j) in the target matrix becomes 0, and the entry (j, i) also becomes 0”.

Only input 1 is taken to form the matrix and adhere to transitivity if it appears in constructing the matrix of the structure of self-interaction. The Transitive Logic states that:

• A has a relationship with B (written as A → B), and
• B has a relationship to C (written as B → C), then
• A has a relationship with C (written as A → C or A → B → C).

The output of this step is also known as the conical matrix.

### Determine the Level Partition of the Reachability Matrix

Based on the reachability matrix from step 3, the elements were divided according to the elements’ influence. Reachability and antecedent designation of each element formed the basis of this division. The reachability set contains the element and other elements that influence the attainment of other elements. The antecedent set contains the element and other elements that assist the achievement of this element. The target matrix’s distribution is essential to develop a model by classifying elements based on the level. The determination of the level of the reachability matrix element was calculated through the determination of intersection sets. The junction of both sets is obtained for all elements. Elements at the intersection were equally representative of the highest levels than other activities in the ISM process.

### Development of the Model

The structural model, also termed the digraph, was generated from the final reachability matrix and level partitioning. Following this, the transitivity links were removed, and numbers were replaced by statements, which led to/created the ISM model.

#### Classification of the Elements (Safety Behavioral Factors) Through Matrice d’Impacts Croises Multiplication Applique a Classement Analysis

By producing the final reachability matrix (conical matrix), Matrice d’Impacts Croises Multiplication Applique a Classement (MICMAC) was used to analyze the driving power and the dependency of the elements to classify them. MICMAC was developed by Duperrin and Godet (1973). This is a systematic analysis tool that classifies variables based on discreet and indirect relationships and assesses how these variables influence and interact. According to Mandal and Deshmukh (1994), the objective of MICMAC analysis is to examine the driver’s power and the co-dependence of variables. “Driving power” is the degree of influence of each variable, “Dependence power” is the extent of influence on one variable by another element (Mandal and Deshmukh, 1994).

The driving power and the dependence of each variable can be obtained from a stable matrix with a sum of 1s in the row and column, respectively. Following this and based on the driver’s power and influence, we can create two-dimensional graphs titled driver dependency diagram. The horizontal axis represents the extent of dependence while the vertical axis the extent of the driver’s power. Using the driver’s power and the dependence of each element, a driver-dependence diagram with four clusters of classification can be constructed: (1) Autonomous Cluster, (2) Independent Cluster, (3) Dependent Cluster, and (4) Cluster Relation (Mandal and Deshmukh, 1994). Cluster details are described below:

(1) Autonomous cluster: Poor driving power and weak, dependent power. There is somewhat disconnected from the system.

(2) Independent cluster: Strong driving power but weak, dependent power. Activities that have a very powerful driving force are called “Main activity.”

(3) Dependent cluster: Low driving power but high dependent power.

(4) Linkage Cluster: Powerful drive power and strong dependent power. These activities are unstable in the fact that any action against these activities will affect others as well as the impact of feedback on themselves.

## Results

### Findings for Step 1

Table 1 presents the ranking of safety behavior factors that were agreed upon by the panel of experts, which should be included in developing the employee safety behavior management model in the downstream oil and gas companies of Malaysia.

**TABLE 1:** Ranking of safety behavior factors.

Ranking	Safety behavior factors
1	Leadership toward safety at the workplace
2	Management commitment to maintaining safety
3	Employees’ response (communication and feedback) about safety at the workplace
4	Employees’ commitment toward safety at the workplace
5	Employees’ attitudes toward safety at the workplace
6	Employees’ awareness toward safety at the workplace
7	Safety training provided for the employees’
8	Employees’ knowledge about safety at the workplace
9	Rules and regulations for safety at the workplace
10	Teamwork toward safety at the workplace
11	Employees’ motivation toward safety at the workplace
12	Employees’ safety involvement at the workplace
13	Safety-related facilities at the workplace
14	Appointment of competent safety-related key personnel
15	Safety Promotion Policies
16	Workplace pressure
17	Establishment of Behavioral Safety (BS) Committee
18	Safety review for continuous improvement

### Findings for Step 2- the Structural Self Interaction Matric

The elements (behavioral safety factors) and a SSIM were developed based on the pair-wise procedure, as shown in Table 2.

**TABLE 2:** Structural self-interaction matrix.

	Safety behavior factors	1	2	3	4	5	6	7	8	9	10	11	12	13	14	15	16	17	18
1	Leadership toward safety at WP		A	V	V	V	V	O	A	X	V	V	V	O	O	V	O	V	V
2	Management commitment to MS			V	V	V	V	V	V	V	V	V	V	V	V	V	V	V	V
3	Employees’ resp. (C&F) to safety at WP				V	V	A	A	A	A	V	V	X	A	A	A	A	A	V
4	Employees’ Commt. toward safety at WP					A	A	A	A	A	V	X	A	A	A	A	A	A	V
5	Employees’ attitudes toward safety at WP						A	A	A	A	V	V	A	A	A	A	A	A	V
6	Employees’ Aware. toward safety at WP							A	A	A	V	V	V	A	A	V	O	A	V
7	Safety training provided for employees’								O	O	V	V	V	V	O	V	O	V	V
8	Employees’ knowledge about safety at WP									V	V	V	V	O	O	V	V	O	V
9	Rules and regulations for safety at WP										V	V	V	O	O	V	O	V	V
10	Teamwork toward safety at WP											A	A	A	A	A	A	A	V
11	Employees’ Motiv. toward safety at WP												A	A	A	A	A	A	V
12	Employees’ safety involvement at WP													A	A	A	A	A	V
13	Safety related facilities at WP														O	V	O	O	V
14	App. of competent safety related personnel															V	O	O	V
15	Safety Promotion Policies																A	A	V
16	Workplace pressure																	O	V
17	Estab. of Behavioral Safety Commt.																		V
18	Safety review for continuous improvement																		

*WP, workplace; MS, maintaining safety; C&F, communication and feedback; Resp, response; Commt, commitment; Aware, awareness; Motiv., motivation; App, appointment; Estab, establishment.*

### Findings for Step 3- Determine the Final Reachability Matrix

By replacing the symbols V, A, X, and O respectively with the binary inputs ‘1’ and ‘0’ as instructed in the methodology section, Table 3 show the final reachability matrix of the relationships among the factors determined collectively by the experts.

**TABLE 3:** Final reachability matrix (conical matrix).

SF	1	2	3	4	5	6	7	8	9	10	11	12	13	14	15	16	17	18	DP
**1**	1	0	1	1	1	1	0	0	1	1	1	1	0	0	1	0	1	1	12
**2**	1	1	1	1	1	1	1	1	1	1	1	1	1	1	1	1	1	1	18
**3**	0	0	1	1	1	0	0	0	0	1	1	1	0	0	0	0	0	1	7
**4**	0	0	0	1	0	0	0	0	0	1	1	0	0	0	0	0	0	1	4
**5**	0	0	0	1	1	0	0	0	0	1	1	0	0	0	0	0	0	1	5
**6**	0	0	1	1	1	1	0	0	0	1	1	1	0	0	1	0	0	1	9
**7**	0	0	1	1	1	1	1	0	0	1	1	1	1	0	1	0	1	1	12
**8**	1	0	1	1	1	1	0	1	1	1	1	1	0	0	1	1	0	1	13
**9**	1	0	1	1	1	**1**	**1**	**0**	**1**	**1**	**1**	**1**	**0**	**0**	**1**	**0**	**1**	**1**	12
**10**	0	0	0	0	0	**0**	**0**	**0**	**0**	**1**	**0**	**0**	**0**	**0**	**0**	**0**	**0**	**1**	2
**11**	0	0	0	1	0	**0**	**0**	**0**	**0**	**1**	**1**	**0**	**0**	**0**	**0**	**0**	**0**	**1**	4
**12**	0	0	1	1	1	**0**	**0**	**0**	**0**	**1**	**1**	**1**	**0**	**0**	**0**	**0**	**0**	**1**	7
**13**	0	0	1	1	1	1	0	0	0	1	1	1	1	0	1	0	0	1	10
**14**	0	0	1	1	1	**1**	**0**	**0**	**0**	**1**	**1**	**1**	**0**	**1**	**1**	**0**	**0**	**1**	10
**15**	0	0	1	1	1	**0**	**0**	0	0	1	**1**	**1**	**0**	**0**	**1**	**0**	**0**	**1**	8
**16**	0	0	1	1	1	**0**	**0**	0	0	1	**1**	**1**	**0**	**0**	**1**	**1**	**0**	**1**	9
**17**	0	0	1	1	1	1	0	0	0	1	1	1	0	0	1	0	**1**	**1**	10
**18**	0	0	0	0	0	0	0	0	0	0	0	0	0	0	0	0	0	1	1
**DEP**	4	1	13	16	14	9	3	2	4	17	16	13	3	2	11	3	5	18	

*SF, safety behavior factors; DP, driving power; DEP, dependence power.*

*If the entry (i, j) in SSIM is V, the entry (i, j) in the target matrix becomes 1, and the entry (i, j) becomes 0*

*If the entry (i, j) in SSIM is A, the entry (i, j) in the target matrix becomes 0, and entry (i, j) becomes 1*

*If the entry (i, j) in SSIM is X, the entry (i, j) in the target matrix becomes 1 and the entry (i, j) also becomes 1, and*

*If the entry (i, j) in SSIM is O, the entry (i, j) in the target matrix becomes 0, and the entry (j, i) also becomes 0.*

### Findings for Step 4- Level Partition of the Reachability Matrix

Based on the findings from Table 3, the level partitioning of the reachability matrix is shown in Table 4.

**TABLE 4:** Level partitioning of the reachability matrix.

Safety factors	Reachability set	Antecedent set	Inter-section set	Level
1	1,3,4,5,6,9,10,11,12,15,17,18	1,2,8,9	1,9	3
2	1,2,3,4,5,6,7,8,9,10,11,12,13,14,15,16,18	2	2	1
3	3,4,5,10,11,12,18	1,2,3,6,7,8,9,12,13,14,15,16,17	3,12	7
4	4,10,11,18	1,2,3,4,5,6,7,8,9,11,12,13,14,15,16, 17	4,11	9
5	4,5,10,11,18	1,2,3,5,6,7,8,9, 12,13,14,15,16, 17	5	8
6	3,4,5,6,10,11,12,15,18	1,2,6,7,8,9,13,14,17	6	5
7	3,4,5,6,7,10,11,12,13,15,17,18	2,7,9	7	3
8	1,3,4,5,6,8,9,10,11,12,15,16,18	2,8	8	2
9	1,3,4,5,6,7,9,10,11,12,15,17,18	1,2,8,9	1,9	3
10	10,18	1,2,3,4,5,6,7,8,9,10, 12,13,14,15,16, 17	10	10
11	4,11,18	1,2,3,4,5,6,7,8,9,11, 12,13,14,15,16, 17	4,11	9
12	3,4,5,10,11,12,18	1,2,3,6,7,8,9, 12,13,14,15,16, 17	3,12	7
13	3,4,5,6,10,11,12,13,15,18	2,7,13	13	4
14	3,4,5,6,10,11,12,14,15,18	2,14	14	4
15	3,4,5,10,11,12,15,18	1,2,6,7,8,9,13,14,15,16,17	15	6
16	3,4,5,10,11,12,15,16,18	2,8,16	16	5
17	3,4,5,6,10,11,12,15,17,18	1,2,7,9,17	17	4
18	18	1,2,3,4,5,7,8,9,10,12,14,15,16,17,18	18	11

### Findings for Step 5- Model Development

**FIGURE 1:**
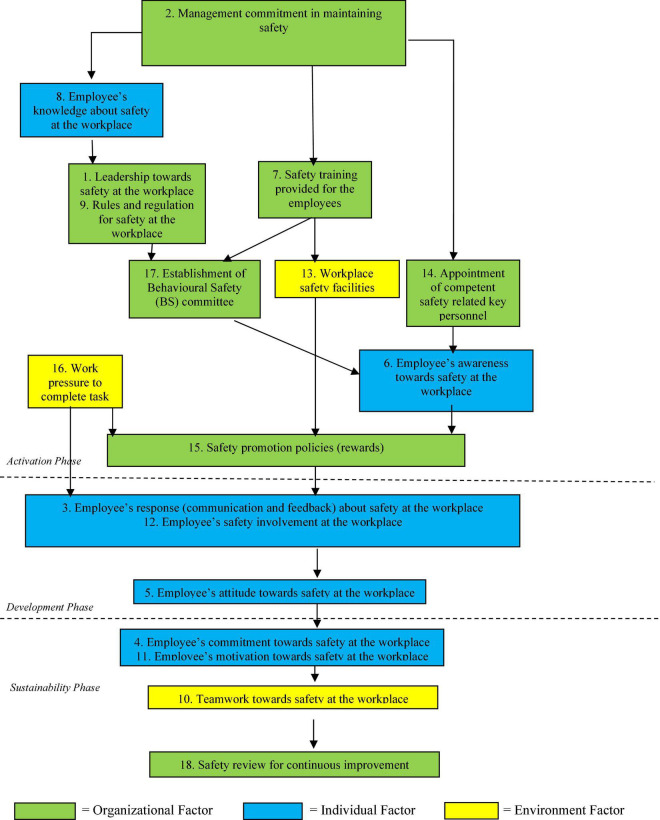
Employee safety behavioral model for downstream oil and gas.

Figure 1 shows the Safety Behavioral Model model based on the findings from steps 1 to 4. The model served as a proposed guide for managing safety behavior among the downstream oil and gas industry employees.

After deliberation of the model, the experts suggested an enhancement be included. The experts indicated that the model could be divided into three phases, which were the activation phase, development phase, and outcome and sustainability phase (as shown in Figure 1). The activation phase consisted of all factors that help activate or trigger the employees’ safety behavior. The development phase comprised all the factors that aid in developing safety behavior among the employees. Based on the model in Figure 1, in terms of the most pertinent factors, the experts had agreed that organizational factors 2, 7, and 14 are crucial in activating the foundation development of safety behavior among the employees. Only one factor under “individual factor” (factor 8) served as part of the activating factors together with these organizational factors. These leading factors lead to subsequent organizational, individual, and environmental factors in this activation phase. The development phase only consisted of individual factors (factors 3, 5, and 12). The sustainability phase comprised all three types of factors (refer to Figure 1).

Referring to the contextual and relation phrase in Step 2 and the model in Figure 1, the arrows showed the flow from one factor to other factors as a set of factors to form an overall structure in implementing the safety behavior management model. For example, factors 2, 8, 7, and 14 need to be managed first before factors 1, 9, and 13. The factors that shared the same box mean that the factors could be managed in any sequence or concurrently as the pair of factors complement each other.

### Step 6- Matrice d’Impacts Croises Multiplication Applique a Classement Analysis

**FIGURE 2:**
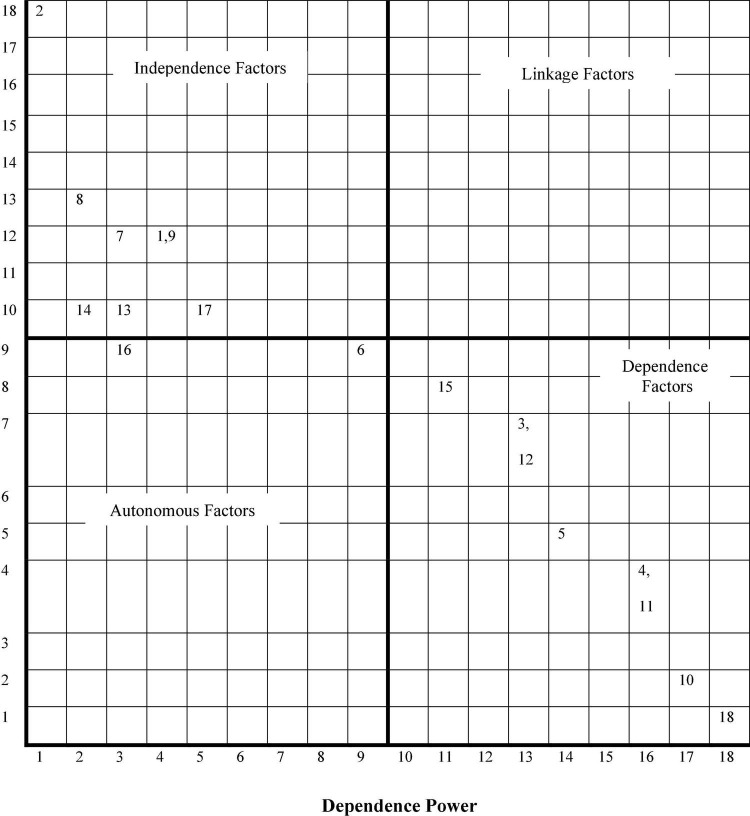
Clusters of variables for safety behavioral model.

As mentioned in the methodology section, MICMAC analysis was conducted to analyze further the degree of influence of the elements (behavioral safety factors). Figure 2 illustrates the MICMAC analysis that reveals how the behavioral safety factors were categorized based on their driving and dependence power. This is summarized in Table 5.

**TABLE 5:** Characteristics of safety behavior factors.

	Cluster	Characteristics	Factors
1	Independent variable	Important factors that need to be considered before other factors	1,2,7,8,9,13,14,17
2	Linkage variable	Factors that serve as the link between Independent and dependent variables	None
3	Autonomous variable	Important factors but somewhat detached from other factors	6,16
4	Dependent variable	Factors which serve as subsequent factors to further develop safety behavior	3,4,5,10,11,12,15,18

## Discussion

Three pertinent categories of factors influencing employees’ safety behavior at the workplace are reflected in the model, namely, organizational, environmental, and individual. As illustrated in Figure 1, the model demonstrated a network and sequence in managing safety behavior among the employees. The model showed the central role of “Management commitment to maintaining safety” in fostering positive safety behavior among the employees. There are theoretical arguments in the literature that management’s commitment to maintaining workplace safety contributes positively to workplace safety behavior. In our study, we extended this theoretical framework and tested it empirically. Hence concluded that supportive management, or a committed leader, builds a work environment where team members feel open to express disagreements, voice opinions, or acknowledge mistakes. Management commitment to safety is a precursor toward achieving positive communication, feedback, and involvement among employees.

The positive work atmosphere fosters employees’ commitment, motivation, and teamwork on safety, which results in progressive safety behavior. Leadership toward safety at the workplace or empowering leadership is highlighted as a useful antecedent of employees’ attitudes and behaviors (Gracia et al., 2020). Prior research suggests that complete reliance on safety rules and regulations is not sustainable to guarantee workplace safety. Supportive leadership through dialogue and open communication promotes a positive safety climate. Employees will be more involved and committed to safety compliance (Martínez-Córcoles et al., 2014). The association between supportive leadership, safety participation, and safety compliance is also supported by Martínez-Córcoles et al. (2014).

Figure 1 shows that factor 2 (management commitment in maintaining safety) is highest in the activation and overall models. This factor is one of the most preliminary factors that need to be managed before other dependent factors. This was also consistent with the findings from MICMAC analysis (Figure 2), where factor 2 was categorized highest in the independent variable cluster, which indicates that it has very high driving power in influencing other variables. This finding is supported by Rajaprasad and Chalapathi (2015), according to which management commitment must be realized before behavioral safety changes among the employees could be obtained.

Other than the management commitment factor, there are additional factors at the organizational and individual levels, such as safety training for employees, appointing key safety personnel, and ensuring employee knowledge about safety procedures. These three factors have high driving and low dependence powers, meaning that these aspects strongly influence other safety behavioral factors. In Figure 1, the factor ‘safety review for continuous improvement’ emerged. This factor is also part of the organizational factors. We conclude that enhanced safety behavior among employees plays a vital role in achieving organizational sustainability. Our research participants helped categorize the model into these three phases: activation, development and outcome, and sustainability. These divisions are also supported by the Antecedent-Behavior-Consequences (ABC) model. Antecedent, also known as an activator, is a trigger that precedes a specific behavior (Scott et al., 2014). Antecedents include triggers, such as equipment, facilities, place, or a person who triggers a specific behavior (Scott et al., 2014). It also suggested further examples of safety antecedents, such as tools, knowledge, skills, rules, regulations, information, signs, and training. Behavior is an action that employees perform to ensure safety and deal with consequences. It is an event that occurs after a certain behavior.

Policymakers must carefully balance the need to stop the spread of the disease with the need to make a better workplace environment. For that the role of both parties, i.e., supervisor and subordinates are important. During the discussion with the experts, it was significantly highlighted that management commitment in maintaining safety is extremely important such as – In hazardous work environment, all employees must be made aware of workplace policies, practices and available support services in general and particularly COVID-19 related information. Appropriate and useful information should be imparted in a timely manner to all employees. Similarly, subordinates need to show their commitments toward safety at the workplace. Besides wearing facemasks and maintaining social distancing at all times, they need to monitor themselves for signs and symptoms of the disease and report their supervisor immediately in case they are experiencing symptoms.

The current research included all the factors suggested by Scott et al. (2014) in the activation phase. However, the model incorporates other elicited factors from the experts, which improves existing models. Additional factors represent the Malaysian downstream oil and gas industry’s suitability stream and focus on various safety behaviors. The antecedents we shared in this model are more specific as they represent an industry. Three main themes were followed for factor elicitation, namely, organizational, individual, and environmental. All these factors are interrelated in the phase of safety behavior development. The findings support the Social Cognitive Theory, where employees, their environment, and their behaviors are related reciprocally. It explains that employees’ behaviors are self-motivated and, at the same time, shaped by their surroundings (You, 2010). As shown in the model, commitment, attitude, awareness, and motivation toward safety will be driven successfully through the surrounding (environmental) and individual itself. The findings also supported (You, 2010), where they stated that employees’ safety behaviors were influenced by the organization that shaped individual cognitions. The Safety Behavior Management Model derived from this study provided evidence that excellent safety behaviors are an amalgamation of organizational, environmental, and individual factors. We focus our attention on using the stakeholder theoretic lens in this article to address organizational sustainability.

The current study employed a holistic model of stakeholder engagement. According to this concept, an organization could gain long-term sustainability by creating value for all stakeholders, without resorting to trade-offs (You, 2010). The list of organizational stakeholders typically includes employees, suppliers, financiers, communities, and customers on understanding the stakeholder relationship of “employees” in the context of safety behaviors. Referring to the model, to manage good and positive safety behavior among the employees, the companies need to focus on trigger factors, then assess the development phase and respond to outcomes. This is done to sustain appropriate safety behavior and gain organizational sustainability. In the model, trigger factors are characterized in three antecedents: organizational (management commitment, safety training, competent person, behavioral safety committee, leadership toward safety, rules, and regulations); individual (safety knowledge); and environmental (workplace safety facilities). All these three antecedents help in triggering safe behavior in employees. The similarity between the ABC Model and ESBM Model is summarized in Table 6.

**TABLE 6:** Similarity between ABC model and safety behavior factors.

ABC model classification	ESBM model classification	Factors
Antecedent	Activation phase	● Management commitment to maintaining safety ●Leadership toward safety at the workplace ● Safety training provided for the employees ● Appointment of competent safety-related key personnel ● Rules and regulation of safety at the workplace ● Establishment of behavioral safety (BS) committee ● Employees’ knowledge about safety at the workplace ● Workplace safety-related facilities
Behavior	Development phase	● Employees’ awareness toward safety at the workplace ● Employees’ response (communication and feedback) about safety at the workplace ● Employees safety involvement at the workplace ● Employees’ attitudes toward safety at the workplace ● Safety promotion policies ● Work pressure to complete task
Consequences	Sustainability phase	● Employees commitment toward safety at the workplace ● Employees’ motivation toward safety at the workplace ● Teamwork toward safety at the workplace ● Safety review for continuous improvement

## Conclusion

Considering the World Health Organization and International Labor Organization reports, there are many reviews and guidance documents about COVID-19 that have been amplified by the COVID-19 pandemic and different experts in the form of certain opinions. The promotion of safety management in the downstream oil and gas industry is complex, as it involves many safety factors. Understanding the intricacies of these factors is pertinent for managing safety behavior to sustain excellent safety performance. This paper highlights a list of factors influencing downstream oil and gas industry safety management in Malaysia based on literature review and in-depth focus group discussions through the NGT. ISM was employed to establish the mapping of interrelationships between these factors that created the Safety Behavior Management Model.

Reflecting on the model, an effort to inculcate safety behavior must begin with a management commitment to maintaining safety where primary prevention of COVID-19 among the workers’ health and safety should be based on risk assessment and introduction of appropriate measures. This needs to be reinforced with a suitable organizational infrastructure that comprises the systems, processes, policies, and operating procedures. This includes sufficient safety training, safety training, competent persons’ supervision, and strategies for information sharing, safety evaluation, and continuous quality improvement. These factors contribute significant latent influences toward the employees’ positive responses, involvement, commitment, teamwork, constructive attitude, and behavior toward safety.

As this safety environment level is achieved, regulating and controlling will be evaded, and sustainable safety behavior can be guaranteed. This research contributed to the improvement of downstream oil and gas industry safety management in Malaysia. The model also shows step-by-step actions that provide practical guidance for developing and implementing strategies to inculcate safety behavior management among the employees. Most factors indicated in the ISM model cannot be accomplished quickly, thus integrating with the companies’ long-term strategy. The ISM analysis helps give the industry practitioners ideas, especially in the downstream oil and gas industry, to consider the impact of the factors on other factors and how to manage safety behavior factors to comply with corporate sustainability practice.

## Data Availability Statement

The raw data supporting the conclusions of this article will be made available by the authors, without undue reservation.

## Author Contributions

All authors listed have made a substantial, direct, and intellectual contribution to the work, and approved it for publication.

## Conflict of Interest

YZ was employed by the company Hoarded Fortune Group, Singapore. The remaining authors declare that the research was conducted in the absence of any commercial or financial relationships that could be construed as a potential conflict of interest.

## Publisher’s Note

All claims expressed in this article are solely those of the authors and do not necessarily represent those of their affiliated organizations, or those of the publisher, the editors and the reviewers. Any product that may be evaluated in this article, or claim that may be made by its manufacturer, is not guaranteed or endorsed by the publisher.
